# Supplementary material for: Comparing in vitro cytotoxic drug sensitivity in colon and pancreatic cancer using 2D and 3D cell models: Contrasting viability and growth inhibition in clinically relevant dose and repeated drug cycles
Source: Cancer Med. 2024 Jun 13;13(11):e7318. doi: 10.1002/cam4.7318 (PMC11176582; doi:10.1002/cam4.7318)
Supplement: Supplementary file 3 — Data S3: [file CAM4-13-e7318-s004.docx]

Additional File 3

// StackProfileData2

// This ImageJ macro gets the profile of all slices in a stack

// and writes the data to the Results table, one column per slice.

//

// Version 1.0, 24-Sep-2010 Michael Schmid

// Version 2.0, 24-Mar-2021 Tia Tidwell

// + batching over image in a folder:

// prompt for selection and autosave results

macro "Stack Profile Data" {

//Displays Prompt for selection of Input & Output Directory

Idir = getDirectory("Choose Input Directory ");

Odir = getDirectory("Choose Output Directory");

list = getFileList(Idir);

if (getVersion>="1.40e")

setOption("display labels", true);

setBatchMode(true);

for (i=0; i<list.length; i++) {

showProgress(i, list.length);

processFile(Idir, Odir, list[i]);

}

function processFile(Idir, Odir, filename)

{

setBatchMode(false);

open(Idir + filename);

run("Stack to Images");

run("Images to Stack", "name=Stack title=[] use");

title = "WaitForUserSeelection";

msg = "Make a line or rectangle selection, then click \"OK\".";

waitForUser(title, msg);

if (!(selectionType()==0 || selectionType==5 || selectionType==6))

exit("Line or Rectangle Selection Required");

run("Plot Profile");

Plot.getValues(x, y);

run("Clear Results");

for (i=0; i<x.length; i++)

setResult("x", i, x[i]);

close();

n = nSlices;

for (slice=1; slice<=n; slice++) {

showProgress(slice, n);

setSlice(slice);

profile = getProfile();

sliceLabel = toString(slice);

sliceData = split(getMetadata("Label"),"\n");

if (sliceData.length>0) {

line0 = sliceData[0];

if (lengthOf(sliceLabel) > 0)

sliceLabel = sliceLabel+ " ("+ line0 + ")";

}

for (i=0; i<profile.length; i++)

setResult(sliceLabel, i, profile[i]);

}

setBatchMode(false);

updateResults;

selectWindow("Results");

saveAs("Results", ""+Odir + filename + "Results.txt");

run("Close All");

}

}
