# Supplementary material for: Comparing in vitro cytotoxic drug sensitivity in colon and pancreatic cancer using 2D and 3D cell models: Contrasting viability and growth inhibition in clinically relevant dose and repeated drug cycles
Source: Cancer Med. 2024 Jun 13;13(11):e7318. doi: 10.1002/cam4.7318 (PMC11176582; doi:10.1002/cam4.7318)
Supplement: Supplementary file 4 — Data S4: [file CAM4-13-e7318-s001.docx]

Additional File 4

// StackProfileData 3.3

// This ImageJ macro gets the profile of all slices in a stack

// and writes the data to the Results table, one column per slice.

//

// Version 1.0, 24-Sep-2010 Michael Schmid

// Version 3.2, 07-Apr-2021 Tia Tidwell

// specific to exported data with stack for each channel

// --load 3 channel files first-- run for each set of stacks

// data output matches same structure as 2.0 macro

// Version 3.3, 09-Apr-2021: added set scale dialog

macro "Stack Profile Data" {

Odir = getDirectory("Choose Output Directory");

Dialog.create("Image position# to select windows for channel merging")

Dialog.addString("Position #:", 01);

Dialog.show();

pos = Dialog.getString();

Dialog.create("Type and Set Scale")

Dialog.addNumber("Number of pixels/um", 0.44);

Dialog.show();

n = Dialog.getNumber();

run("Set Scale...", "distance=n known=1 pixel=1 unit=um global");

selectWindow("Position0" + pos + "_RAW_ch00.tif");

run("Stack to Images");

selectWindow("Position0" + pos + "_RAW_ch01.tif");

run("Stack to Images");

selectWindow("Position0" + pos + "_RAW_ch02.tif");

run("Stack to Images");

run("Images to Stack", "name=Stack title=[] use");

title = "WaitForUserSeelection";

msg = "Make a line or rectangle selection, then click \"OK\".";

waitForUser(title, msg);

if (!(selectionType()==0 || selectionType==5 || selectionType==6))

exit("Line or Rectangle Selection Required");

run("Plot Profile");

Plot.getValues(x, y);

run("Clear Results");

for (i=0; i<x.length; i++)

setResult("x", i, x[i]);

close();

n = nSlices;

for (slice=1; slice<=n; slice++) {

showProgress(slice, n);

setSlice(slice);

profile = getProfile();

sliceLabel = toString(slice);

sliceData = split(getMetadata("Label"),"\n");

if (sliceData.length>0) {

line0 = sliceData[0];

if (lengthOf(sliceLabel) > 0)

sliceLabel = sliceLabel+ " ("+ line0 + ")";

}

for (i=0; i<profile.length; i++)

setResult(sliceLabel, i, profile[i]);

}

setBatchMode(false);

updateResults;

selectWindow("Results");

saveAs("Results", ""+ Odir + "Pos" + pos + "_results.txt");

run("Close All");

}
